# The mechanism of *ATF3* repression of epithelial‐mesenchymal transition and suppression of cell viability in cholangiocarcinoma via *p53* signal pathway

**DOI:** 10.1111/jcmm.14132

**Published:** 2019-01-16

**Authors:** Zhen You, Jingchang Xu, Bei Li, Hui Ye, Liping Chen, Yang Liu, Xianze Xiong

**Affiliations:** ^1^ Department of Biliary Surgery West China Hospital of Sichuan University Chengdu Sichuan China; ^2^ Ambulatory Surgery Center West China Hospital of Sichuan University Chengdu Sichuan China

**Keywords:** *ATF3*, cholangiocarcinoma, EMT, *p53* signalling pathway

## Abstract

The aim of this research was to determine the underlying mechanism of activating transcription factor 3 (*ATF3*) on cell proliferation, invasion, migration and epithelial‐mesenchymal transition (EMT). The differentially expressed mRNAs in cholangiocarcinoma (CC) and its adjacent tissues were screened by microarray analysis, and the expression of ATF3 was detected through Quantitative real time polymerase chain reaction (qRT‐PCR) and Western blot. The expression of EMT markers and *p53‐*related proteins was analysed by Western blot. Analyses using the Cell Counting Kit‐8 and TUNEL were performed to assess the rate of apoptosis and cell proliferation. Scratch wound and transwell assays were performed to study cell migration and invasion. Activating transcription factor 3 was restrained in CC cell lines and tissues and inhibited EMT while activating the *p53* signalling pathway. Knockdown of *ATF3* promoted cell proliferation but reduced the rate of apoptosis by inhibiting *p53* signalling. Cell migration and invasion can be strengthened by *ATF3 *through activating the *p53 *signalling pathway.

## 1 INTRODUCTION

Human cholangiocarcinoma (CC) arises from the epithelium of the biliary tree, including adenocarcinomas located in the gall bladder and the intra‐ or extrahepatic biliary tree.^1^ The etiology of CC may be correlated with the presence of bile duct stones and primary sclerosing cholangitis. Treatments for CC include surgery, radiotherapy and chemotherapy, but prognosis remains poor.^2^ Even with aggressive surgical treatment, CC patients remain subject to a high post‐operative recurrence rate between 67% and 75%.^3^ Thus, there is an important and urgent need for new biomarkers to improve early diagnosis and reduce the rate of extrahepatic CC.^4^

Epithelial‐mesenchymal transition (EMT) is a significant biomarker for cancer diagnosis. Expression of the epithelial cell marker E‐cadherin is up‐regulated while expression of the mesenchymal markers vimentin and N‐cadherin were inhibited in transformed cells.^5^ Proteins such as Snail1 and Twist bind to the promoter of E‐cadherin to suppress expression.^6^ Recent evidence highlights the critical role of EMT not only in promoting cancer metastasis and immune escape but also in the progression of CC.^3^

The immediate early gene activating transcription factor 3 (*ATF3*) is an *ATF*/*CREB* family member whose expression is rapidly induced by a wide range of cellular stresses including DNA damage, cellular injury and oxidative stress.^7^ Recent studies indicated that *ATF3* is strongly associated with cancer development.^8^ Depending on the tumour type, *ATF3* may induce tumour cell apoptosis or improve tumour cell survival.^9^ Xin et  al verified that *ATF3* enhances EMT in breast cancer cells,^10^ while other studies revealed that *ATF3* plays a tumour suppressing role in many different cancer types, including colon cancer, esophageal squamous cell carcinoma (ESCC) and hepatocellular carcinoma (HCC).^9^, ^11^ Very little research has directly addressed the role of *ATF3* in human CC; therefore, we hypothesized that *ATF3* might repress the process of EMT to suppress the development of CC.

*p53*, a downstream target of ATF3, was the first tumour suppressor gene discovered and can inhibit cancer cells through multiple pathways, such as promoting cancer cell apoptosis and restraining the cell cycle.^2^ *p53* is primarily regulated by the E3 ubiquitin ligase Murine Double Minute 2 (*MDM2*), which binds *p5*3 at its transactivation domain to block *p53*‐mediated transcriptional regulation while simultaneously promoting polyubiquitination and proteasome‐dependent degradation of p53.^12^ Recent studies have reported that *ATF3* can modulate the activity of *p53*.^13^ A central leucine zipper domain (Zip) in *ATF3* mediates protein‐protein interactions. Activating transcription factor 3 and *p53 *are bound through Zip, stimulating *p53* tumour inhibitor activity independent of *ATF3* transcription.^14^ The aim of our study was to investigate the effects of *ATF3* on cell viability via activating the *p53* signalling pathway.

This research explored the differentially expressed mRNAs in CC compared to its adjacent tissues and analysed the expression of *ATF3*, EMT markers and *p53*‐related proteins. Cell migration, the apoptosis rate, proliferation and invasion were also analysed in this study. We found that ATF3 repressed EMT and restrained cell migration, proliferation and invasion while enhancing cell apoptosis via activating *p53* signalling.

## 2 MATERIALS AND METHODS

### 2.1 Clinical specimens

Ten pairs of human bile duct tissues and adjacent tissues were obtained after informed consent was provided from patients at the West China Hospital of Sichuan University between September 2015 and March 2017. Normal and CC specimens were obtained from patients with R0 surgically resected bile ducts. The protocols used in the study adhere to regulations established by the Ethics Committee of the West China Hospital of Sichuan University.

### 2.2 Cell culture and treatment

The human bile duct intrahepatic epithelial cell line HIBEpiC, the human CC intrahepatic cell lines HuCCT1 and RBE and the human hilar CC cell line QBC939 were obtained from BeNa Culture Collection (Beijing, China). The human hilar CC cell line FRH0201 was purchased from Huayun Biotech (Guangzhou, China). HuCCT1 and QBC939 were cultivated in Dulbecco's modified Eagle's medium (DMEM; Gibco BRL, Grand Island, NY, USA) supplemented with 10% FBS (Gibco BRL), penicillin G (10^5^ U/L) and streptomycin (100 mg/L; Gibco BRL) in a humidified atmosphere containing 5% CO_2_ at 37°C. Groups of cells were treated with the MDM2 inhibitor/*p53* agonist MX69 (MedChemExpress, Monmouth Junction, NJ, USA).

### 2.3 Microarray analysis

The gene expression profiles of eight pairs of tumour tissues and adjacent tissues (seven pairs of stage I‐II, one pair of stage III‐IV) obtained from The Cancer Genome Atlas (TCGA) (https://cancergenome.nih.gov/) were analysed in this study. Differentially expressed mRNAs between normal and cancerous bile duct specimens were screened using the significance analysis of microarrays (SAMR) package in r software, and |log_2_ fold change (FC)| > 2 and false discovery rate < 0.05. Cluster analysis was then performed to confirm whether the identified mRNAs could be used to robustly classify normal and CC specimens.

### 2.4 Cell transfection

Two* ATF3* siRNAs (si‐*ATF3*) and an *ATF3*‐pcDNA 3.1 plasmid were purchased from Gene Pharma (Shanghai, China) and were packaged by 293 T cells to generate virus. QBC939 and FRH0201 cells were seeded at 10^5^ cells per well in 24‐well plates during logarithmic growth and cultured until 80% confluence. Cells were transfected with the corresponding lentivirus using Lentifectin^TM^ (ABM, Richmond, BC, Canada) according to the manufacturer's recommendations. Fluorescent microscopy was used for determining the transfection efficiency 48 hours post transfection.

### 2.5 qRT‐PCR

RNA from both tissues and cells was extracted using Trizol reagent (Beyotime, Shanghai, China) according to the manufacturer's guidelines. The PrimeScript^TM ^RT reagent kit (Takara, Tokyo, Japan) was used to generate cDNA. qPCR was performed using SYBR Premix Ex Taq™ GC (Takara) and an Applied Biosystems 7500 real‐time PCR system (Applied Biosystems, Foster City, CA, USA). *GAPDH* was used as an internal control for *ATF3* and repeated in triplicate. Samples were normalized to internal controls, and FCs were obtained using the 2^−ΔΔCT^ method. The primer sequences used are listed in Table 1.

**Table 1:** Primers for qRT‐PCR

Genes		Primer sequence 5′‐3′
α‐catenin	F	GCTTCGGGCCTCTGGAATTT
	R	ATGTTGCCTCGCTTCACAG
E‐Cardherin	F	GAACTCAGCCAAGTGTAAAA
	R	GAGTCTGAACTGACTTCCGC
Vimentin	F	TCCGCACATTCGAGCAAAGA
	R	ATTCAAGTCTCAGCGGGCTC
Fibronetin	F	AACTTCCTGGTGCGTTACTCA
	R	TGTGCTCTCATGTTGTTCGT
Snail1	F	AGGACCCCACATCCTTCTCA
	R	GCACCTGGGGGTGGATTATT
Slug	F	ACAGCGAACTGGACACACAT
	R	TTGCCGCAGATCTTGCAAAC
Twist	F	CTTCTCGGTCTGGAGGATGG
	R	GCACGACCTCTTGAGAATGC
p53	F	ACCCAGGTCCAGATGAAG
	R	GCAAGAAGCCCAGACG
MDM2	F	AGTTGCGCTTTATGGGTGGA
	R	TGAGTACAGCAATCATTTCAGATGC
Bax	F	CCAAGAAGCTGAGCGAGTGT
	R	CGTCCTGGAGACAGGGACAT
p21	F	GCAGACCAGCATGACAGATTTC
	R	CTTCCTGTGGGCGGATTAGG
PUMA	F	GACCTCAACGCACAGTACGA
	R	GAGATTGTACAGGACCCTCCA
ATF3	F	TGGCAACACGGAGTAAACGA
	R	GCATCATTTTGCTCCAGGCTC
GAPDH	F	CGGAGTCAACGGATTTGGTCGTAT
	R	AGCCTTCTCCATGGTGGTGAAGAC

ATF3, activating transcription factor 3; MDM2, Murine Double Minute 2; PUMA, p53 up‐regulated modulator of apoptosis.

### 2.6 Western blot

Briefly, cells were washed with phosphate buffered saline (PBS) before harvesting with lysis buffer (Sangon Biotech, Co., Ltd., Shanghai, China). Protein was separated by SDS‐PAGE and transferred to a Polyvinylidene Fluoride (PVDF) membrane. Non‐specific binding was blocked with Tris Buffered Saline Tween (TBST) containing 5% (w/v) non‐fat dried milk. The membrane was then incubated with rabbit anti‐ATF3 (1:1000; Abcam, Cambridge, MA, USA), rabbit anti‐α‐catenin (1:50000; Abcam), rabbit anti‐E‐cadherin (1:10000; Abcam), rabbit anti‐Vimentin (1:2000; Abcam), rabbit anti‐Fibronectin (1:1000; Abcam), rabbit anti‐p53 (1:1000; Abcam), rabbit anti‐MDM2 (1:1000; Abcam), rabbit anti‐Bax (1:1500; Abcam), rabbit anti‐Slug (1:1000; Abcam), rabbit anti‐Snail1 (1:1000; Abcam), rabbit anti‐Twist (0.5 μg/mL, Abcam) and rabbit anti‐GAPDH (1:500; Abcam) at 4°C overnight. Next, membranes were incubated overnight with an Horseradish Peroxidase (HRP)‐conjugated goat anti‐rabbit IgG (1:10000; Abcam) for 1 hour at 37°C. The membranes were developed with an Emitter‐Coupled Logic (ECL) chemiluminescence detection kit and quantitated using ImageJ software. GAPDH served as a loading control.

### 2.7 Cell Counting Kit‐8 assay

Transfected cells (3 × 10^3^) were seeded in 96‐well plates in a final volume of 100 μL per well and cultured for 24, 48, 72, and 96 hours. Cell Counting Kit‐8 (CCK‐8) solution (10 μL) was added to each well. Cells were then cultured at 37°C for 4 hours. After incubation, the absorbance at 450 nm was measured using a Multiskan FC microplate reader (Thermo Fisher Scientific, Waltham, MA, USA) to calculate the number of viable cells. The experiment was independently repeated three times.

### 2.8 TUNEL assay

We seeded 2 × 10^5^ cells in a 24‐well plate and incubated them for 24 hours. After fixation with 4% paraformaldehyde, the cells were infiltrated in 3% hydrogen peroxide/methanol for 10 minutes. Cells were then permeabilized with 0.5% Triton for 5 minutes, incubated with 50 μL of TUNEL reaction liquid and incubated for 1 hour at 37°C. The cells were imaged using the EVOS FL fluorescence microscope (Thermo Fisher Scientific), and five fields of view were selected for quantification. The apoptosis rate was quantitated as the TUNEL‐positive cell number.

### 2.9 Scratch wound assay

Six‐well plates were seeded with 10^5^ cells, cultured until 60% confluency, and then scratched with a plastic filter tip to make a “wound”. Cells were washed three times with PBS and cultured at 37°C. After 0 and 48 hours, the average distance that the cells migrated was measured with an inverted microscope. ImageJ software was used to calculate the migration distance. Migration area (%) = ([area of 0 hour − experimental group area of 48 hours)/area of 0 hour)*100. The experiments were carried out in triplicate.

### 2.10 Transwell assay

The Transwell chamber was coated with 80 μL of Matrigel (BD Biosciences, San Jose, CA, USA) hydrated in 50 μL of serum‐free medium. Next, a 200‐μL suspension of transfected cells (1 × 10^5 ^cells/mL) was added to the upper chamber, and media containing 30% FBS was added to the bottom chamber. After culturing for 24 hours, cells were fixed for 15 minutes in 4% formaldehyde and stained with 1% crystal violet. Cells were counted and the experiment repeated in triplicate.

### 2.11 Statistical analysis

Data are represented as the mean ± SD and analysed by GraphPad Prism 6.0 software (GraphPad Software, La Jolla, CA, USA). Statistical differences of treatment groups were calculated using ANOVA and Student's *t* test. *P < *0.05 was considered statistically significant.

## 3 RESULTS

### 3.1 *ATF3* is expressed at a low level in CC cell lines and tissues

**Figure 1:**
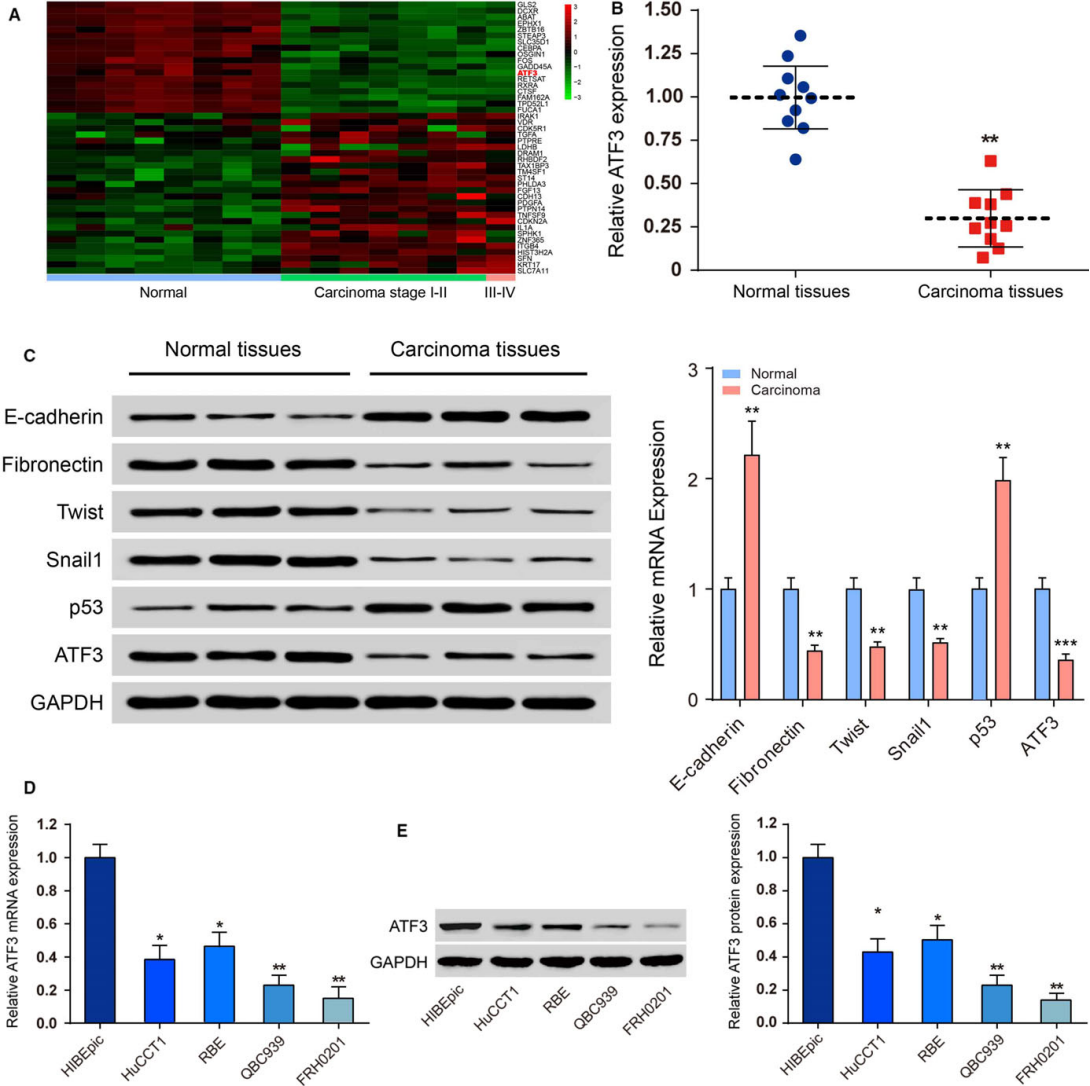
Activating transcription factor 3 (ATF3) was down‐regulated in bile duct tumour tissues. A, The heat map of differentially expressed mRNAs in normal and cholangiocarcinoma (CC) specimens showed that the expression level of ATF3 was reduced in stage I‐II and III‐IV CCs. B, Quantitative real time polymerase chain reaction (qRT‐PCR) was performed to confirm the expression of ATF3 in normal and CC tissues. **P < 0.01. C, Western blot (left) and qRT‐PCR (right) were conducted to determine protein and mRNA levels of epithelial‐mesenchymal transition markers and p53 in normal and CC tissues, respectively. D, qRT‐PCR was utilized to determine the mRNA level of ATF3 in immortalized human bile duct epithelial cells (HIBEpic) and CC cell lines (HuCCT1, RBE, QBC939 and FRH0201). *P < 0.05, **P < 0.01. E, Western blotting results revealed that ATF3 protein level was reduced in CC cell lines (HuCCT1, RBE, QBC939 and FRH0201) compared to bile duct epithelial cells (HIBEpic). *P < 0.05, **P < 0.01

**Figure 2:**
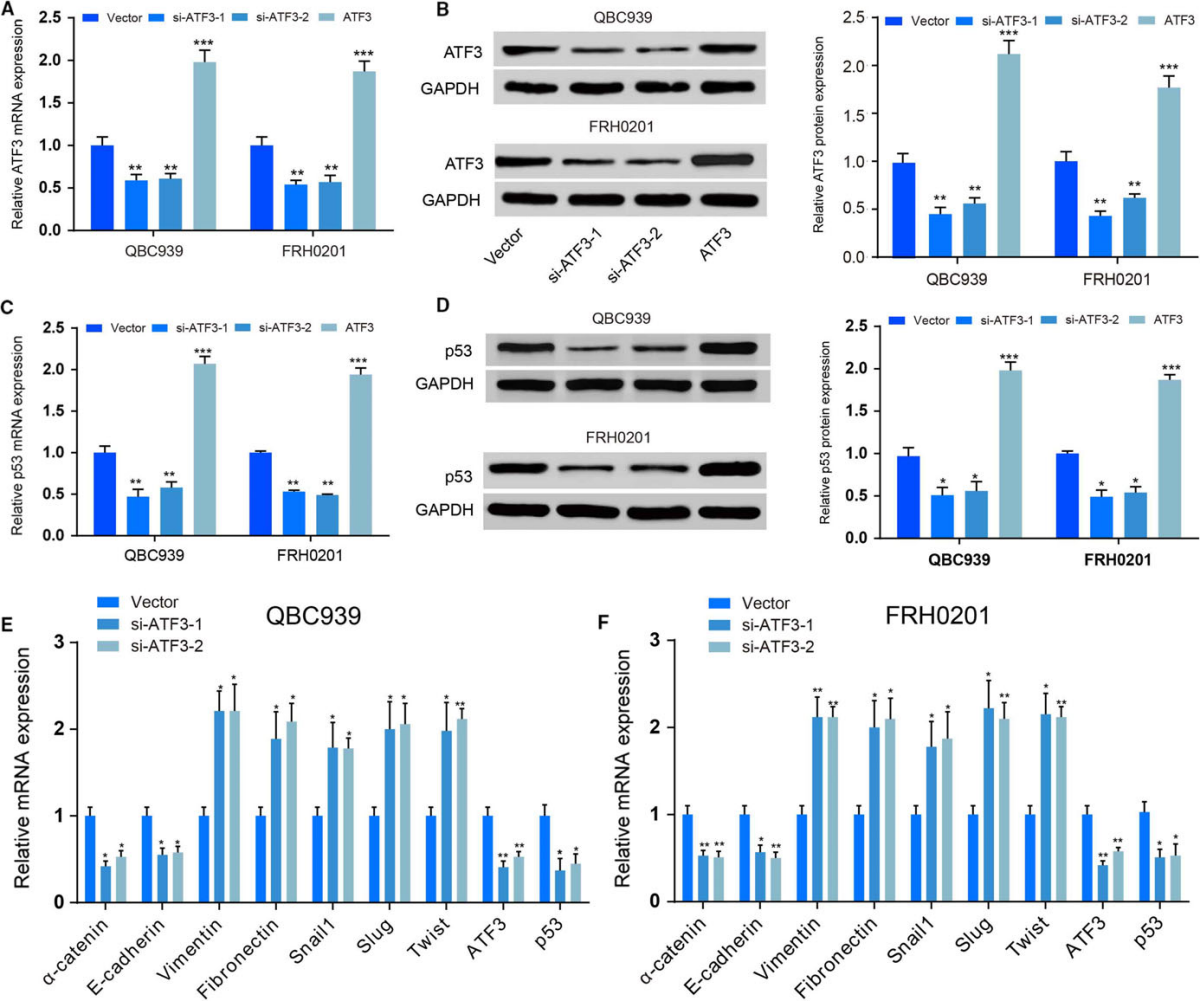
Effects of si‐ATF3 and ATF3‐pcDNA 3.1 on activating transcription factor 3 (ATF3) expression A, QBC939 and FRH0201 cells were transfected with ATF siRNAs or ATF3‐pcDNA 3.1 for 48 h, and then, qRT‐PCR was performed to determine knockdown efficiency. **P < 0.01. B, Western blot showed that the protein expression of ATF3 was inhibited in QBC939 and FRH0201 cells transfected with si‐ATF3. **P < 0.01, compared with the vector‐only control group. Activating transcription factor 3 protein was overexpressed in QBC939 and FRH0201 cells transfected with ATF3‐pcDNA 3.1. ***P < 0.001, compared with the vector‐only control group. C, QBC939 and FRH0201 cells were transfected with ATF siRNAs or ATF3‐pcDNA 3.1 for 48 h, and then, qRT‐PCR was performed to determine mRNA level of p53. **P < 0.01. D, QBC939 and FRH0201 cells were transfected with ATF siRNAs or ATF3‐pcDNA 3.1 for 48 h, and then, Western blot was performed to determine protein level of p53. **P < 0.01. (E,F) qRT‐PCR were conducted to determine the mRNA levels of EMT markers in QBC939 and FRH0201 cells. *P < 0.05, **P < 0.01.

According to the microarray analysis, *ATF3* was markedly repressed in different CC tissues (Figure 1A). The expression of *ATF3* was markedly decreased in CC tissues compared with normal tissues, as detected by qRT‐PCR (*P < *0.01, Figure 1B). Figure 1C indicates that EMT‐ and p53‐associated proteins were significantly altered in CC tissues. In addition, both mRNA expression (*P < *0.01, Figure 1D) and protein expression (*P < *0.01, Figure 1E) of *ATF3* were significantly lower in the four human CC cell lines compared with the bile duct epithelial cell line HIBEpic, which verified that *ATF3* was not highly expressed in CC cells. The QBC939 and FRH0201 cell lines were transfected with both si‐ATF3 and ATF3‐pcDNA3.1. The relative expression of *ATF3* mRNA and protein was analysed by Western blot as well as qRT‐PCR *ATF3* expression was significantly down‐regulated after knockdown (*P < *0.01) compared to QBC939 and FRH0201 cells (*P < *0.01, Figure 2). These results suggested that si‐*ATF3* inhibited the expression of *ATF3*, whereas *ATF3*‐pcDNA 3.1 promoted *ATF3* expression in CC cells; thus, the transfection was successful.

### 3.2 ATF3 suppresses EMT in CC cells

**Figure 3:**
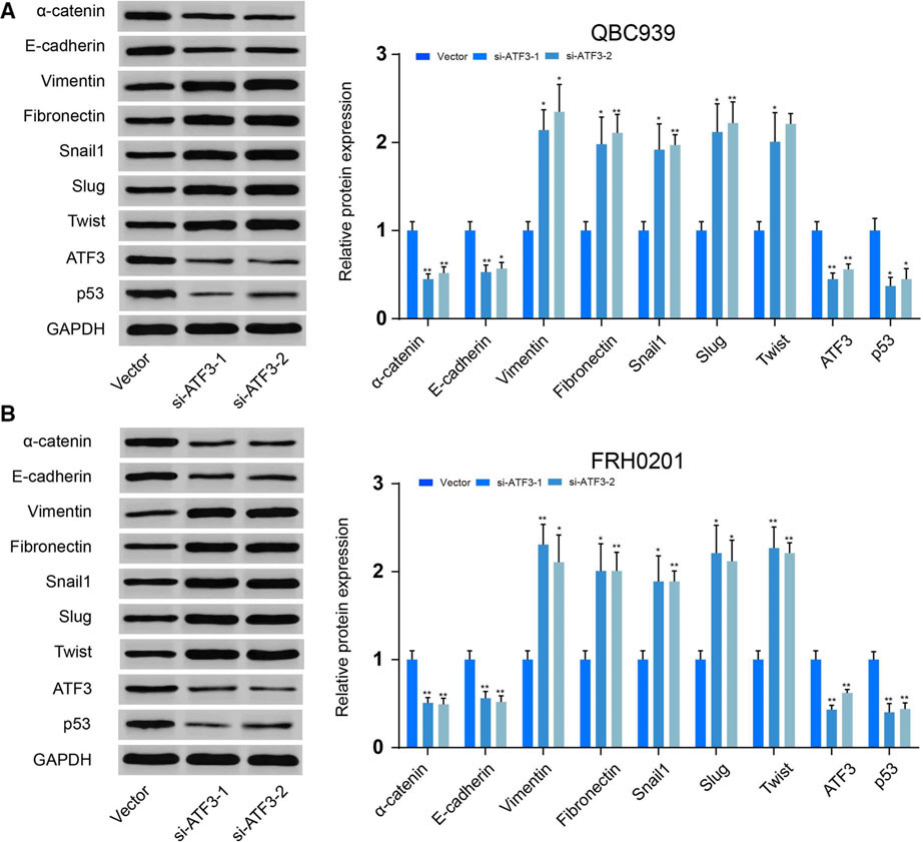
Activating transcription factor 3 (ATF3) suppresses epithelial‐mesenchymal transition (EMT) in cholangiocarcinoma cells (A,B) QBC939 (A) and FRH0201 (B) cells were transfected with ATF siRNAs for 48 h, and then, Western blot were conducted to examine the protein levels of EMT markers (α‐catenin, E‐cadherin, Vimentin, Fibronectin, Snail1, Slug, Twist, ATF3) and p53. *P < 0.05, **P < 0.01.

**Figure 4:**
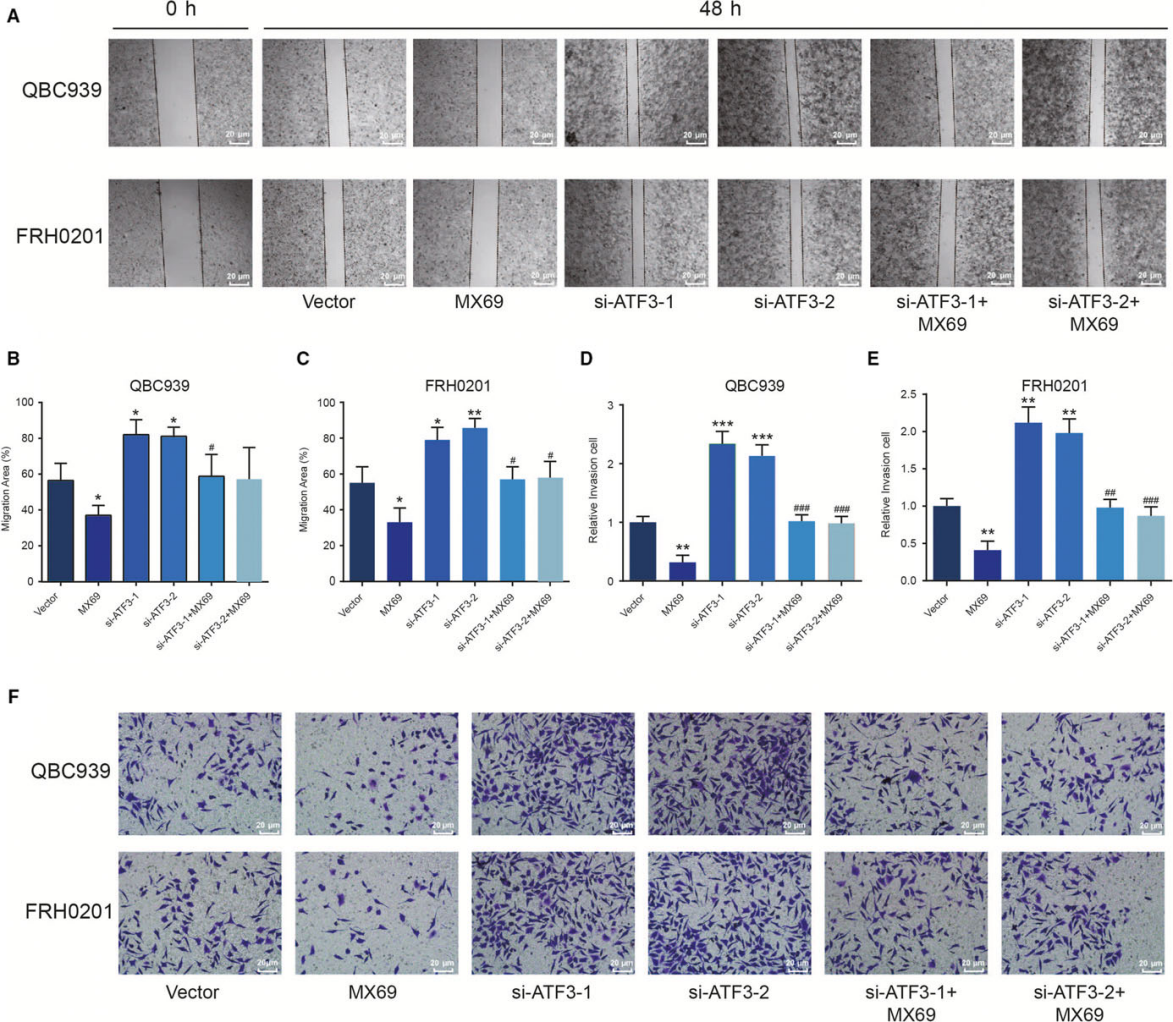
Activating transcription factor 3 (ATF3) suppresses cell migration and invasion (A‐C) The scratch wound assay revealed that cell the migration capacity of QBC939 and FRH0201 cells in the MX69 group was decreased but was increased in the si‐ATF3‐treated group. The migration capacity was not significantly different between the MX69 and si‐ATF3‐treated groups. *P < 0.05, **P < 0.01, compared with the vector group; #*P < *0.05, ##*P < *0.01, compared with the siRNA groups. (D‐F) The Transwell assay showed that MX69 inhibited invasion of QBC939 and FRH0201 cells and that knockdown with si‐ATF3 had the opposite effect. The invasion capacity was not significantly different in the MX69 and si‐ATF3 groups. **P < 0.01, ***P < 0.001, compared with the vector group; #*P < *0.05, ##*P < *0.01, ###*P* < 0.001, compared with the siRNA‐treated groups.

The epithelial markers α‐catenin and E‐cadherin showed robust down‐regulation, while the expression of the mesenchymal markers Vimentin and Fibronectin increased after down‐regulation of *ATF3* (*P < *0.01, Figure 2D‐F). Transcriptional expression of the three canonical EMT markers Snail1, Slug and Twist was up‐regulated after *ATF3 *knockdown (*P < *0.01, Figure 2D‐F), which was further confirmed with similar western blotting results (Figure 3). Scratch wound and Transwell assays also demonstrated that MX69 inhibited the cell invasion and migration ability of QBC939 and FRH0201 cells, whereas si‐*ATF3* had an opposite effect that did not change in the MX69 and si‐*ATF3* group (*P < *0.01, Figure 4). From these experiments, we conclude that *ATF3* can partially repress EMT in CC cells.

### 3.3 ATF3 activates the *p53* signalling pathway

**Figure 5:**
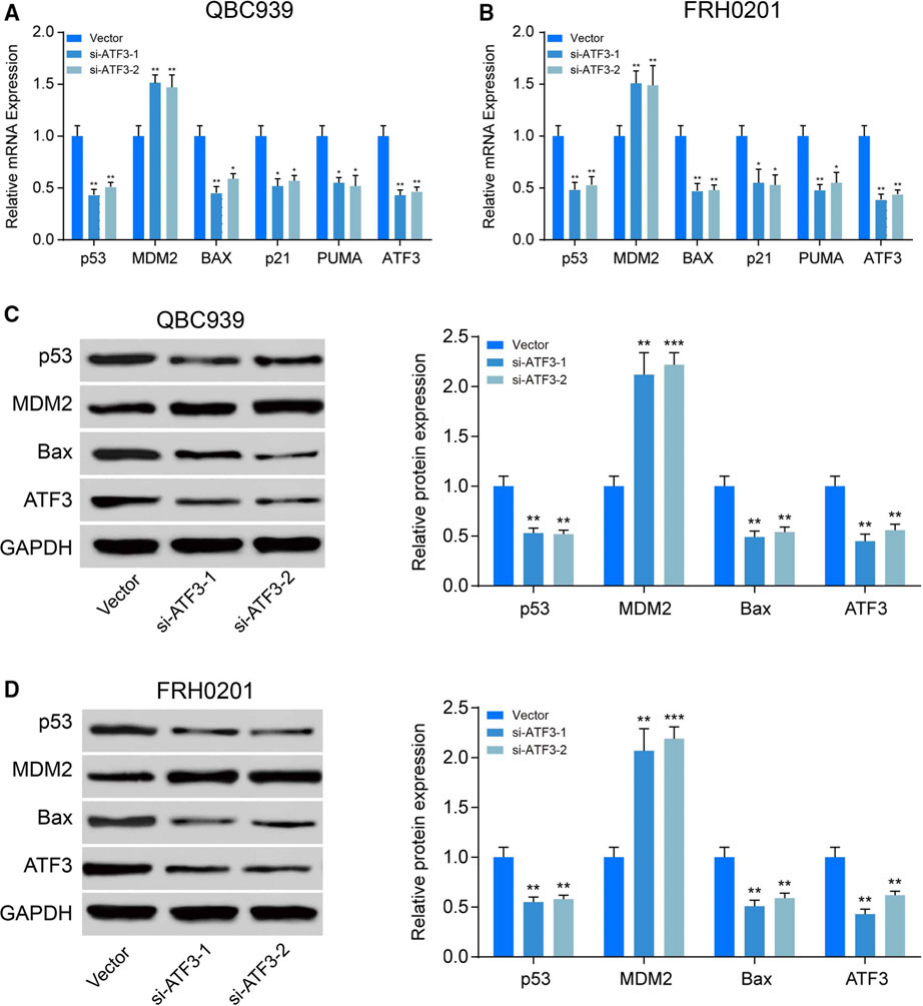
Activating transcription factor 3 (ATF3) activates the *p53* signalling pathway (A) QBC939 cells were transfected with ATF siRNAs for 48 h, and then, qRT‐PCR was performed to determine mRNA levels of p53 and its target genes such as Bax, p21 and p53 up‐regulated modulator of apoptosis (PUMA). **P < 0.01. B, FRH0201 cells were transfected with ATF siRNAs for 48 h, and then, qRT‐PCR was performed to determine mRNA levels of p53 and its target genes such as Bax, p21 and PUMA. **P < 0.01. C, QBC939 cells were transfected with ATF siRNAs for 48 h, and then, Western blot was performed to determine protein levels of p53 and its target genes such as Bax, p21 and PUMA. **P < 0.01. D, FRH0201 cells were transfected with ATF siRNAs for 48 h, and then, Western blot was performed to determine protein levels of p53 and its target genes such as Bax, p21 and PUMA. **P < 0.01.

qRT‐PCR assays indicated that ATF3 knockdown might inhibit the expression of *p53* and its target genes such as *Bax*, *p21* and p53 up‐regulated modulator of apoptosis (PUMA) (*P < *0.01, Figure 5A,B). Western blotting indicated that low expression of *ATF3* might repress the expression of *p53* and the downstream proapoptotic protein *Bax.* MDM2 antagonized *p53* activity and was highly expressed in both QBC939 and FRH0201 cells (*P < *0.01, Figure 5C,D). Collectively, these results indicated that *ATF3* activates the *p53* signalling pathway and therefore affects the progression of CC.

### 3.4 ATF3 suppresses cell viability via the *p53* signalling pathway

**Figure 6:**
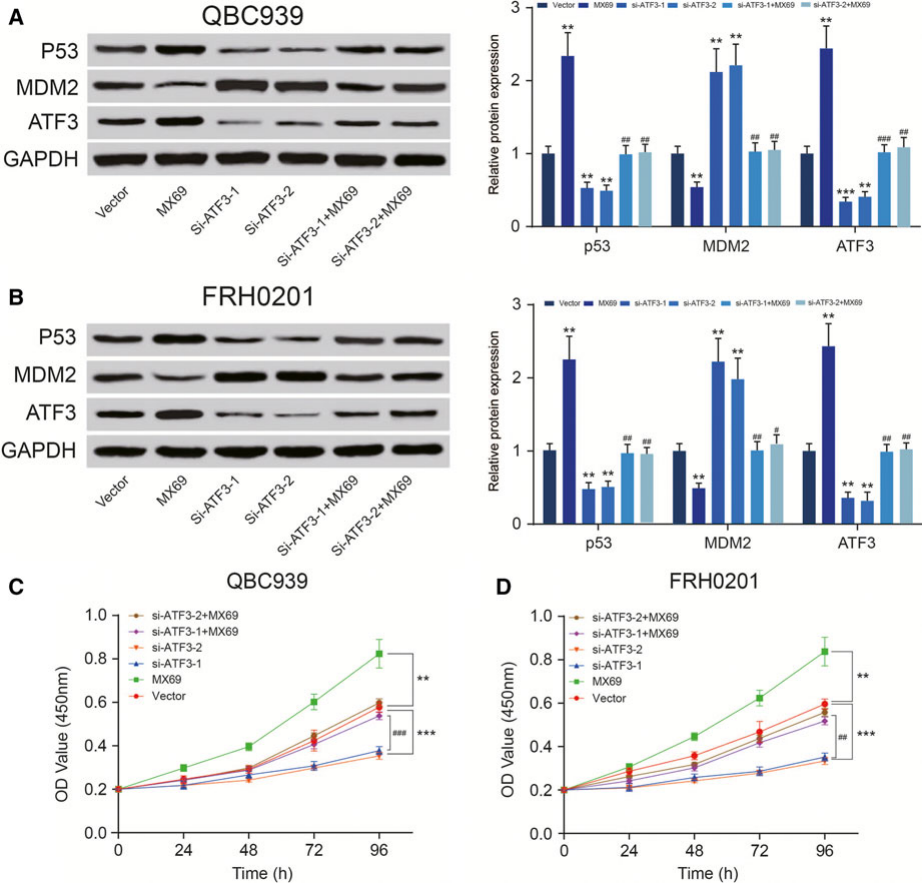
Activating transcription factor 3 (ATF3) suppresses cell proliferation and increases the apoptosis rate of cholangiocarcinoma cells (A,B) Western blot showed that MX69 up‐regulated ATF3 and *p53* expression in QBC939 and FRH0201 cells and down‐regulated MDM2, while siATF‐1 and siATF‐2 transfection yield opposite results. Expression levels of ATF3, *p53* and MDM2 were not significantly different in the MX69 + si‐ATF3 groups and vector group. *P < 0.05, **P < 0.01, compared to the vector‐only control group; ##*P < *0.01, compared to the siRNA‐treated groups. (C,D) Cell Counting Kit‐8 assay were performed to determine the proliferation capacity of QBC939 and FRH0201 cells subjected to MX69 treatment or si‐ATFs transfection or MX69+si‐ATFs treatment. *P < 0.05, **P < 0.01, ***P < 0.001, compared with the vector‐only control group; ##*P < *0.01, compared with the siRNA‐treated groups.

**Figure 7:**
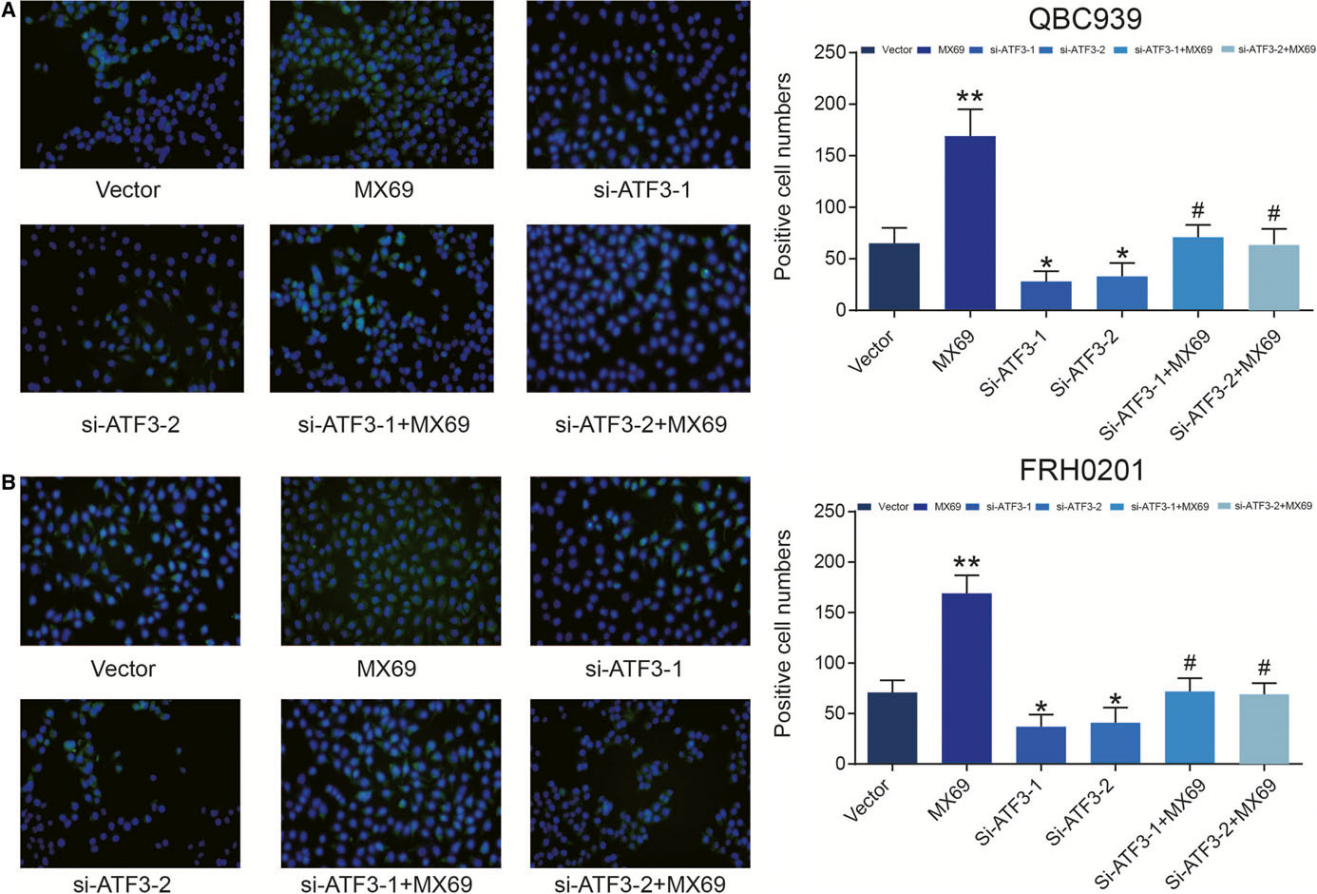
TdT‐mediated dUTP Nick‐End Labeling (TUNEL) assay (A) Representative TUNEL assay of QBC939 cells and (B) FRH0201 cells subjected to MX69 treatment or si‐ATFs transfection or MX69 + si‐ATFs treatment. MX69 treatment increased apoptosis in QBC939 and FRH0201 cells, and si‐ATF3 treatment inhibited apoptosis. The rate of apoptosis in the MX69 + si‐ATF3‐treated groups was not significantly different from the vector‐only control group. **P < 0.01, compared to the vector‐only control group; ##*P < *0.01, compared to the siRNA‐treated groups.

**Figure 8:**
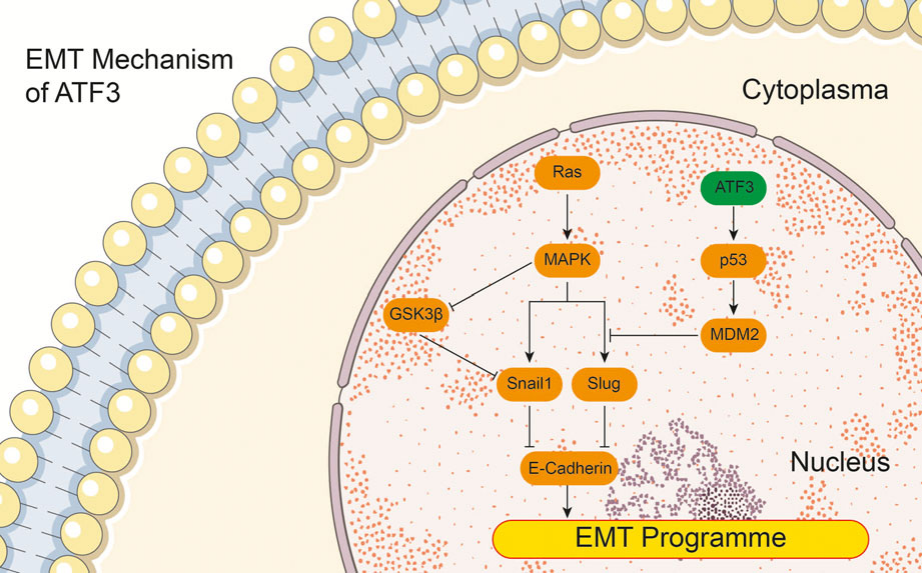
Schematic of the potential mechanism of activating transcription factor 3 (ATF3) in epithelial‐mesenchymal transition (EMT) of cholangiocarcinoma (CC) cells. Activating transcription factor 3 dysregulation might affect p53 expression. Because Murine Double Minute 2 is a p53 target, it can also inhibit the expression of Slug, which is an EMT gene. Thus, dysregulation of Slug might disrupt expression of E‐Cadherin, further disrupting EMT in CC cells. The orange rounded rectangles indicate the genes involved in EMT, and the green rounded rectangle indicates ATF3

MX69 (*MDM2* suppressor/*p53* activator) markedly enhanced expression of *p53*, while si‐*ATF3 *inhibited expression of *p53*. However, *p53* expression levels did not significantly change when cells were cultured with MX69 and si‐*ATF3* (*P < *0.01, Figure 6A,B). The CCK‐8 assay revealed that MX69 inhibited proliferation of QBC939 and FRH0201 cells in contrast to the si‐*ATF3*‐treated group. Cell proliferation was almost identical in the vector‐only control group after MX69 or si‐*ATF3* treatment (*P < *0.01, Figure 6C,D). Similarly, the TdT‐mediated dUTP Nick‐End Labeling (TUNEL) assay illustrated that QBC939 and FRH0201 cells transfected with MX69 exhibited a higher apoptosis rate than the control group. Although si‐*ATF3* decreased the rate of apoptosis, there was no observable change in cells treated with both MX69 and si‐*ATF3* (*P < *0.01, Figure 7). In conclusion, ATF3 inhibits cell invasion, proliferation and migration while increasing the apoptosis of cancer cells in the bile duct via the *p53* signalling pathway. The potential mechanism of ATF3 in EMT is shown in Figure 8.

## 4 DISCUSSION

We observed that *ATF3* expression was decreased in CC cell lines and tissues and that it inhibited EMT while activating the *p53* signalling pathway. Knockdown of *ATF3* strengthened cell invasion, proliferation and migration and reduced apoptosis via inhibiting the *p53* signalling pathway.

Epithelial‐mesenchymal transition is an important step in invasion and metastasis. The invasive ability of cancer cells is acquired via transformation to a mesenchymal phenotype, during which the expression of the EMT markers N‐cadherin, E‐cadherin and vimentin changes.^15^ Although *ATF3* promotes EMT in breast cancer, the mechanism of *ATF3* in EMT depends on the tumour type. Overexpression of ATF3 increases expression of N‐cadherin, vimentin and FN but inhibits E‐cadherin expression. *ATF3* knockdown reduced EMT features as indicated by the molecular markers in breast cancer.^10^ This finding contrasts with other studies in which *ATF3* represses EMT in a variety of tumours, including colorectal cancer.^16^ This study revealed the role of *ATF3* in CC, and knockdown of *ATF3* robustly down‐regulated the epithelial markers α‐catenin and E‐cadherin, as well as up‐regulated the mesenchymal markers Vimentin and Fibronectin. This result suggests that *ATF3* represses the process of EMT in CC cells.

We also investigated the interactions between *ATF3* and *p53*. The *P53* gene encodes a tumour suppressor protein that contains DNA binding, transcriptional activation and oligomerization domains.^17^, ^18^ A recent study showed that *ZNF545* acted as a functional tumour suppressor in multiple myelomas via activating the *p53* signalling pathway.^19^ Yan et  al described that activating the *p53* pathway in *CNPY2* knockout cells reduced cell growth, migration, colony formation and angiogenesis but increased cellular apoptosis in colorectal tumours.^20^ Furthermore, a regulatory feedback mechanism between *p53* and *ATF3 *was previously proposed.^21^ Wang et  al found that *ATF3* activated *p53* and thus protected human papillomavirus (HPV)‐infected cervix epithelium against oncogenic transformation in cervical cancers.^22^ Consistent with previous reports, we found that the expression of *p53* and its downstream protein *Bax*, which promotes apoptosis,^23^, ^24^ was inhibited while expression of MDM2, an antagonist of *p53*,^25^ was increased after suppressing *ATF3*. Furthermore, *ATF3* inhibits cell proliferation, invasion and migration while increasing the apoptosis rate of cancer cells of the bile duct via the *p53* signalling pathway.

In summary, the results showed that expression of *ATF3* was clearly inhibited in CC tissues and cell lines and could efficiently inhibit EMT. We also demonstrated that overexpression of *ATF3* repressed cell invasion, proliferation and migration while increasing the apoptosis rate via activating the *p53* signalling pathway.

Our results reveal the molecular mechanism of *ATF3* in the development of CC and provide a new therapeutic target for treatment. However, the study did not investigate the molecular mechanisms of *ATF*3 in EMT. As a result, additional experiments exploring the role of *ATF3* in EMT are required, as well as in vivo experiments. Finally, improvements in experimental methods may help to better distinguish between the rate of proliferation and changes in cell migration and invasion.

## ETHICS APPROVAL

The study was approved by the Ethics Boards of West China Hospital of Sichuan University.

## CONFLICT OF INTEREST

The authors declare that they have no competing interests.

## AUTHOR CONTRIBUTION

Contributing to the conception and design: Zhen You, Jingchang Xu, Liping Chen; Analysing and interpreting data: Bei Li, Hui Ye, Yang Liu; Drafting the article: Zhen You; Revising it critically for important intellectual content: Xianze Xiong; Approving the final version to be published: All authors.
